# Status and outlook for acaricide and insecticide discovery

**DOI:** 10.1002/ps.6084

**Published:** 2020-09-28

**Authors:** Peter Jeschke

**Affiliations:** ^1^ Bayer AG, Research & Development, Crop Science Pest Control Chemistry Monheim am Rhein Germany

**Keywords:** acaricides, structure–activity relationship, insecticides, mode of action, physicochemistry, selectivity, resistance

## Abstract

To guarantee sustainability and progress, the agrochemical industry is faced with several major challenges. Currently, loss of active ingredients due to consumer perception, changing grower needs and ever‐changing regulatory requirements is far higher than the number being introduced into the market. Therefore, there is a need to develop new products that can provide improved efficacy, selectivity and favorable environmental profiles. Strategies to achieve these goals are the search for acaricides and insecticides with new modes of action, or the discovery of novel molecules with activity on the most attractive target sites having resistance breaking properties against pest species. In this context, the introduction of halogen atoms or asymmetric centers into an active ingredient remains an important tool to modulate their properties, but so too is the pro‐pesticide concept. This review gives an overview of agrochemicals launched over the past 8 years, reflects new insights into known mechanisms of action, and describes the status and outlook for acaricide and insecticide discovery.

## Graphical abstract

Since 2008, around 89% of the launched products (one acaricide and eight insecticides) have contained halogen atoms, in most cases fluorine, but also ‘mixed’ halogen atoms, for example chlorine and fluorine. Of these, three insecticides have been launched as racemic mixtures of enantiomers and one as an enantiomerically pure *semi‐*synthetic natural product. In addition, the pro‐pesticide concept has been used.

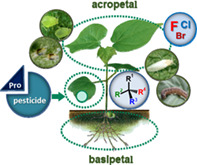

## 1 INTRODUCTION

Effective control of insect pest populations by modern products in agriculture and horticulture must currently correlate with the many features required for optimal efficacy, low application rate in the field, improved selectivity, enhanced user friendliness, favorable toxicological and environmental safety, and protect non‐target organisms. Obtaining products that meet most of these requirements has become the focus of attention for agrochemical and food companies, regulatory authorities, farmers, and the general public. The use of beneficial insects, combined with acaricides and insecticides, new formulation concepts, and versatile application methods (e.g. drone technology or soil and seed treatments used against insect vectors for plant virus diseases) has intensified. The development of effective active ingredients (a.i.) with novel mode of actions (MoAs) and narrower insecticidal spectra (e.g. active only against sucking pest‐targeted or certain chewing pest‐targeted insects) has become a new focus.

Numerous destructive pest arthropod species (mites and insects) have evolved high levels of (cross) resistance against different classes of acaricides and insecticides, either by overexpression of metabolic enzymes to rapidly detoxifying acaricides and insecticides, or by selection for rare mutant alleles conferring target‐site resistance.^1^ This is observed in particular, in several insect species of intense agronomic cropping systems where acaricide and insecticide control options are limited, and where repeated applications of the same MoA target consecutive generations of the same insect pest.

In this context, resistance is considered an extremely serious problem requiring a proactive approach. Therefore, an effective insect resistance management (IRM) strategy is mandatory, and the Insecticide Resistance Action Committee (IRAC) has been dedicated to making this a reality for the past three decades.^2^

**Figure 1:**
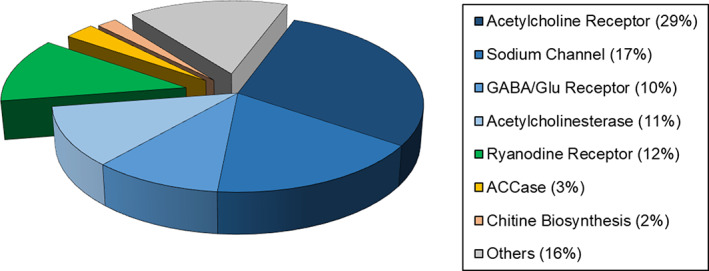
Central nervous system and muscle physiology of insects are common targets for commercial products. The majority of the a.i.s act on nerves (blue) and muscle physiology (green) with fewer compounds acting on respiration, growth and development, midgut, on unknown target or they act non‐specific. Based on distribution of insecticide sales by IRAC MoA main groups on 2018 End‐user sales data (total = $19.8 billion USD) (Agranova, 2019).^3^

The global insecticide market is currently dominated by only six insecticide molecular target sites covering around 79% of the market: nicotinic acetylcholine receptor (*n*AChR), sodium channel (SoCh), γ‐aminobutyric acid‐gated (GABA)‐chloride channel, glutamate‐gated chloride (GluCl) channel, acetylcholinesterase (AChE) and the ryanodine receptor (RyR). As such, only a few receptors, channels or enzymes in the central nervous system (CNS) and muscle physiology of insects are targeted by commercial a.i. (Fig. 1).

With a total market share of 29% in 2019, *n*AChR remains one of the most attractive target sites for pest control. Neonicotinoids (IRAC sub‐group 4A) binding to this target are the most important class of insecticides and their lasting success is reflected in their market share of ~ 24%.^3^

By contrast to insecticides, the target physiology of the main acaricidal classes is different. Based on the distribution of acaricide sales data (AgroWin, 2018), the global acaricide market is currently dominated by six acaricide molecular target sites as exemplified by the frontrunner acaricides: mitochondrial complex II electron transport (MET II) (cyflumetofen, ~ €18.8 T), mitochondrial complex III electron transport (MET III) (bifenazate, ~ €29.5 T), mitochondrial complex I electron transport (MET I) (pyridaben, ~ €31.6 T), an ATP‐synthase (propargite, ~ €42.9 T), acetyl coenzyme A carboxylase (ACCase) (spirodiclofen, ~ €62.1 T) and the glutamate‐gated chloride (GluCl) channel (abamectin, ~ €126.4 T).

The macrolactones as GluCl channel allosteric modulators (IRAC sub‐group 6) and tetronic and tetramic acid derivatives of IRAC sub‐group 23 are the most important classes of acaricides, which are also applied as mixtures with other acaricides with another MoA.

This review provides an overview of the latest generation of acaricides and insecticides brought into the global crop protection market in the past 8 years (Table S1), addresses today's most important MoAs, reflects new insights into known mechanisms of action, and describes the status and prospects for acaricide and insecticide discovery.

## 2 NOVEL MECHANISMS OF ACTION

Progress has been made in finding novel MoAs of acaricides and insecticides that function by affecting the mitochondrial complex II electron transport (MET II), notably via a compound that achieves selectivity through the ‘pro‐acaricide’ concept^4^, ^5^ (Section 2.1), and by modulating the Nan‐Iav vanilloid subtype of transient receptor potential (TRPV) channels (Section 2.2) and by insecticide structures that act as GABA‐gated chloride channel allosteric modulators (Section 2.3).

With the GS‐omega/kappa‐HXTX‐Hv1A spider venom peptide, a new peptide‐based neurotoxin acting as a *n*AChR allosteric modulator acting at site II has been commercialized in 2018 (Section 2.4).

### 2.1 Mitochondrial complex II electron transport inhibitors

#### 2.1.1 *MET* *I and* *MET III* *inhibitors*

Acaricides and fungicides have been discovered from the same chemical group acting as MET inhibitors, such as pyridineamines (MET I) and strobilurins (MET III) at the respiratory chain for example:

1. MET I inhibitors: pyrimidifen (IRAC sub‐group 21A) *versus* diflumetorim (Fungicide Resistance Action Committee (FRAC) target no. C1); and
2. MET III inhibitors: fluacrypyrim (IRAC sub‐group 20C) *versus* kresoxim‐methyl (FRAC target no. C3).

#### 2.1.2 *MET II* *inhibitors*

Based on structural similarity to the fungicidal succinate dehydrogenase (SDH) inhibitor carboxamides, active as MET II inhibitors (FRAC target no. C1), it was concluded that the new carboxanilide acaricide pyflubumide has the same MoA.

**Figure 2:**
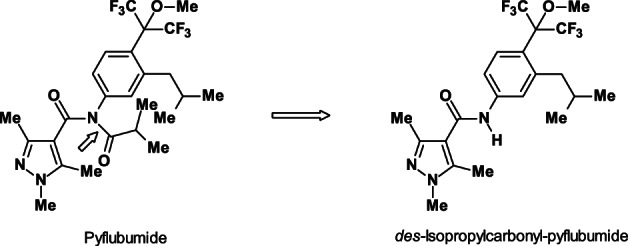
Structures of the pro‐insecticide pyflubumide and its acaricidally active metabolite *des‐*isopropylcarbonyl‐pyflubumide. Arrow shows the cleavage site.

Pyflubumide (Fig. 2) contains a 1‐methoxy‐hexafluoro‐*iso*‐propyl residue^6^ which is structurally inspired by the heptafluoro‐*iso*‐propyl residue from the insecticide flubendiamide^7^ (C(CF_3_)_2_‐F *versus* C(CF_3_)_2_‐OMe).

The branched 3‐*iso*‐butyl side chain at the phenyl group and the 1,3,5‐trimethyl‐pyrazole‐carboxylic acid moiety originated from the SDHI fungicide penthiopyrad (FRAC target no. C2). Structural optimization resulted in replacement of a heptafluoro‐*iso*‐propyl residue by a hexafluoro‐*iso*‐propyl residue which is favorable for acaricidal activity against the two‐spotted spider mite (*Tetranychus urticae*).

Surprisingly, the heptafluoro‐*iso*‐propyl residue results in a sharp decrease in acaricidal activity, because of its high lipophilicity. Conversely, the hexafluoro‐*iso*‐propyl residue leads to the most active acaricide, but the high efficacy was combined with off‐target toxicity. Therefore, the slightly more lipophilic 4‐(1‐methoxy‐hexafluoro‐*iso*‐propyl) residue was introduced, which maintained the acaricidal activity. After *N*‐deacylation of the pro‐acaricide pyflubumide, the formed NH‐metabolite strongly inhibits the MET II, binding in a possibly different manner from the known pro‐acaricidal *beta*‐ketonitrile derivatives^8^ such as cyenopyrafen and cyflumetofen (IRAC MoA sub‐group 24A).^9^ Therefore, pyflubumide has been classified as a carboxanilide in the new IRAC MoA sub‐group 24B.

Pyflubumide is active against globally important polyphagous spider mites, including *Tetranychus* spp. and *Panonychus* spp.^10^, and has good activity against field populations resistant to conventional acaricides such as the MET I inhibitor fenpyroximate (IRAC sub‐group 21A), the MET III inhibitor acequinocyl (IRAC sub‐group 20B) or the mite growth inhibitor etoxazole.^10^ Furthermore, pyflubumide has almost no toxicity against non‐target arthropods, and has low toxicity to mammals and fishes.

Recently, a first resistance risk assessment of pyflubumide has been carried out, providing first evidence for metabolic resistance mediated by cytochrome P_450_ (CYP_450_) in the red spider mite *Tetranychus urticae*.^11^

In addition, target‐site resistance mutations in *T*. *urticae* for the acaricidal SDH inhibitors pyflubumide and cyenopyrafen have been detected by using quantitative trait locus (QTL) analysis.^12^

### 2.2 Chordotonal organ TRPV channel modulators

**Figure 3:**
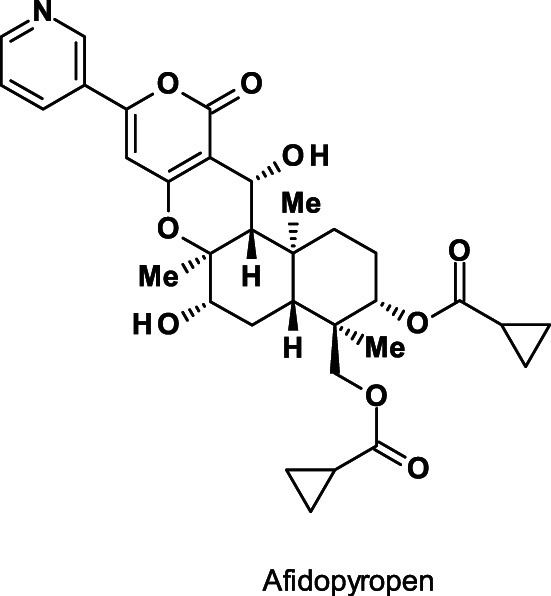
Structure of the insecticide afidopyropen.

Afidopyropen (Fig. 3), is a potent and specific modulator of insect Nan‐Iav vanilloid subtype of TRPV cation channels.^13^

This is a *semi‐*synthetic derivative of the natural product pyripyropen A, which itself is produced by *Aspergillus fumigatus*.^14^ Afidopyropen is potent against sucking insects such as pea aphids (*Acyrthosiphon pisum*)^15^ and has been classified according to IRAC distinct from pyridine azomethine derivatives such as pymetrozine and pyrifluquinazon (MoA sub‐group 9B), as the first member of the novel pyropene class of the MoA sub‐group 9D.

The insecticide afidopyropen acts rapidly with a quick onset of feeding cessation, leading to reduced virus transmission. It exhibits good translaminar movement but is not fully systemic. Therefore, utilizing spray volumes that result in full coverage of plant surfaces is recommended to achieve optimum performance. Afidopyropen provides effective control of devastating piercing and sucking insect pests, such as aphids, whiteflies, leafhoppers, certain psyllids (e.g. Asian citrus psyllid, *Diaphorina citri*) and scales, including those that have developed resistance to other insecticides.

It is relatively non‐toxic to natural enemies and might be considered an effective option for IPM and IRM programs for soybean aphid (*Aphis glycines*).^16^

### 2.3 GABA‐gated chloride channel allosteric modulators

The GABA‐gated chloride channel is an important target for insecticides.^17^ Unfortunately, major target insects have evolved resistance to the first generation of non‐competitive antagonists (NCAs), the so‐called GABA‐gated chloride channel blockers (IRAC MoA main group 2) such as the cyclodienes (IRAC MoA sub‐group 2A; e.g. chlordane, endosulfan). NCAs inhibit the permeation of chloride ions and induce hyper‐excitation, thereby resulting in insect death. However, mutations at the M2 membrane‐spanning region such as A2′S and A2′G reduce NCA antagonist activity.^18^ Both mutations promote cross‐resistance to second‐generation NCAs such as phenylpyrazoles (fiproles) (IRAC MoA sub‐group 2B; e.g. fipronil, ethiprole), although the resistance level is low. Finally, the A2′N mutation reduces the activity of fipronil conferring full resistance to field rates.^19^

In 2018, arylisoxazolines (Section 2.3.2) were recognized by IRAC as the first class of GABA‐gated chloride channel allosteric modulators (IRAC main group 30) causing hyper‐excitation and convulsion. A second class *meta*‐diamides (Section 2.3.2) followed 1 year later.

#### 2.3.1 *Arylisoxazolines*

**Figure 4:**
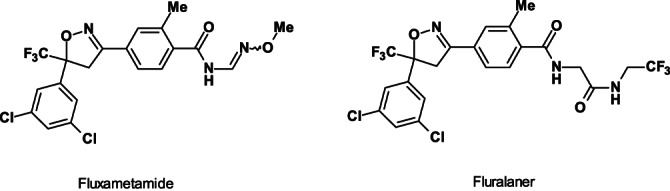
Structures of the insecticide fluxametamide and ectoparasiticide fluralaner.

Fluxametamid (Fig. 4), is a racemic isoxazoline insecticide (contains an isomeric methoxime group) that demonstrates high activity against various lepidopteran, thysanopteran and dipteran pest species.^20^ It is also acaricidal active against tetranychid mites (Acari: *Tetranychidae*).

It appears that in view of the lack of a cost‐efficient asymmetric synthesis technology, it is probable that for the next few years only the racemic mixture of fluxametamide will be produced, although the (*S*)‐enantiomer is quite a bit more active. This economic decision correlates well with the structurally related and commercial racemic isoxazoline fluralaner (Fig. 4).^21^ Only the (*S*)‐enantiomer of the ectoparasiticide fluralaner has activity with no adverse effects caused by the (*R*)‐enantiomer.^22^

Fluxametamide is a ligand‐gated chloride channel (LGCC) antagonist, inhibiting GABA‐gated chloride channels and Glu‐gated chloride channels. Fluxametamide controls both fipronil‐susceptible and ‐resistant arthropod pests at similar rates. Fluxametamide antagonizes arthropod GABA‐gated chloride channels by binding to a site different from those for existing antagonists, which is why it controls fipronil‐resistant insects. Fluxametamide has high target‐site selectivity for arthropods over mammals. The acute toxicity of fluxametamide enantiomers toward honey bees has been evaluated. Its *S*‐(*+*)*‐*isomer exhibited 52.1–304.4 and 2.5–3.7 times higher bioactivity than the *R‐*(−)*‐*isomer and *rac‐*fluxametamide, respectively.^23^ The *S‐*(+)*‐*isomer showed more than 30‐fold higher acute toxicity than the *R‐*(−)‐isomer. Molecular docking studies have been performed with GABA receptor to monitor the mechanism of stereoselective bioactivity.^23^ The separated *R‐*(−)‐isomer of fluxametamide may not be completely insecticidal inactive, but it demonstrates insufficient field performance.

#### 2.3.2 *Selected development candidates active at the* *GABA‐gated chloride channel*

**Figure 5:**
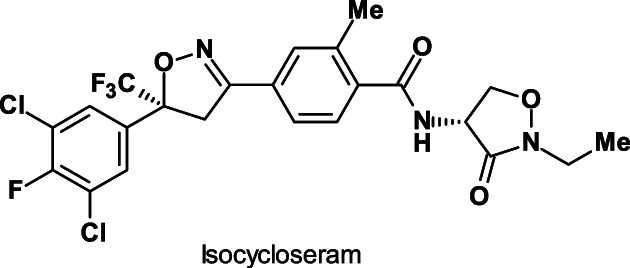
Structure of the insecticide development candidate isocycloseram.

Isocycloseram [containing 80–100% of the (5*S*,4*R*)‐isomer); common name ISO‐provisionally approved; Fig. 5] is a broad‐spectrum arylisoxazoline insecticide and acaricide that is active against lepidopteran, hemipteran, coleopteran, thysanopteran and dipteran pest species.

A cost‐efficient asymmetric technology has been developed to produce the active isocycloseram (5*S*,4*R*)‐diastereomere with an enantiomeric excess.

Similar to fluxametamid (Section 2.3.1), isocycloseram is a GABA‐gated chloride channel allosteric modulator and acts as a non‐competitive GABA‐gated chloride channel antagonist at a site different from known antagonists such as fiproles and cyclodienes, and thus can be used to control resistant insects.

#### 2.3.3 *Meta‐diamides*

**Figure 6:**
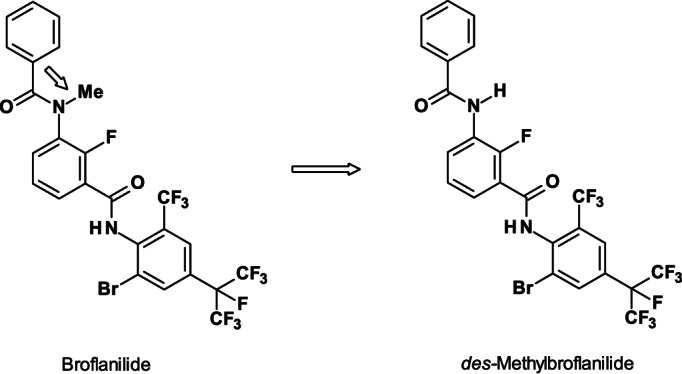
Structures of the pro‐insecticide broflanilide and its insecticidally active metabolite *des‐*methyl‐broflanilide. Arrow shows the cleavage site.

The *meta*‐diamides such as broflanilide (Fig. 6),^24^ were discovered by structural modification of the RyR modulator flubendiamide.^7^ Surprisingly, the change in the amide group from an *ortho* to *meta*‐position resulted in a shift in the MoA (from RyR modulator to a GABA‐gated chloride channel allosteric modulator). Broflanilide contains one bromine and 11 fluorine atoms, located in the 2‐fluoro‐benzamide in addition to a 2‐bromo‐4‐heptafluoro‐*iso*‐pyropyl‐6‐trifluoromethyl‐phenyl as fragments of the molecule.

Structure–activity relationship (SAR) experiments with the *meta‐*diamides demonstrated that the activity against oriental leafworm moth (*Spodoptera litura*) and diamondback moth (*Plutella xylostella*) is preferred if fluorine is in the 2‐position. Conversely, for activity against cotton bollworm (*Helicoverpa armigera*), a combination of bromine, trifluoromethyl group and the strong lipophilic heptafluoro‐*iso*‐propyl substituent has to be present.^25^

Broflanilide has broad‐spectrum activity (crop and non‐crop pests) and can be used in different crops to control lepidoptera, coleoptera, termites, ants, cockroaches, and flies. The pro‐insecticide broflanilide is probably metabolized by CYP_450_‐mediated bioactivation of its insecticidally active *des‐*methyl‐broflanilide (cleavage of the *N*‐methyl group).^8^ This acts as a non‐competitive GABA receptor antagonist on a novel site in *Drosophila* RDL GABA receptors (resistant‐to‐dieldrin, RDL). This site is at or close to G336 in the M3 region of *Drosophila* RDL GABA receptors.^26^ Although the site of action for *meta*‐diamides seems to overlap with that of Glu‐gated chloride channel allosteric modulators, such as avermectins and milbemycins, different MoAs have been discussed.^27^

The high selectivity of the metabolite *des*‐methyl‐broflanilide is probably the reason why broflanilide is effective against pests that evolved resistance to GABA‐receptor channel blocker‐type antagonists such as cyclodienes and phenylpyrazoles (fiproles). The insecticidal activity of broflanilide was not different between susceptible and resistant biotypes carrying A2′S, A2′G, and A2′N mutations in the GABA‐receptor channel in each species.

The insecticidally active metabolite *des*‐methyl‐broflanilide exhibits higher selectivity towards insect RDL GABA receptors in tobacco cutworm (*S*. *litura*) *versus* the human GABA type A receptor (GABA_A_R) α1β2γ2, mammalian (GABA_A_R) α1β3γ2, and human glycine receptor (GlyR) α1β (Table 1).^28^

### 2.4 Nicotinic acetylcholine receptor (*n*AChR) allosteric modulators – site II

The spinosyns, classified as IRAC main group 5 ‘*n*AChR allosteric modulators – site I’, activate *n*AChRs at a site that is allosteric to the orthosteric site where nicotine and its mimics are active. Recently, the IRAC named this nicotinic allosteric modulator site I to differentiate it from *n*AChR allosteric modulator site II. The target site of the new main group 32 consists of the GS‐omega/kappa‐HXTX‐Hv1A *Hadronyche versuta* spider venom peptide (a hexatoxin).^29^ The peptide‐based neurotoxin is commercially produced using a transgenic *Kluyveromyces lactis* yeast. The yeast is considered by the U.S. Food and Drug Administration (FDA) as ‘non‐pathogenic and non‐toxicogenic’.

This ecofriendly neurotoxin is used on fruits, vegetables and high‐value field crops, and has selective contact activity to lepidopteran pests such as cabbage looper caterpillars, loopers as well as larvae of beetles, moths, and thrips, without negatively affecting beneficial arthropod species, such as pollinators, and is non‐toxic to bees, fish and mammals. Targeted insect pests die within hours after oral ingestion or physical contact with the neurotoxic protein. Application may be made by ground, air, or chemigation.

## 3 APPROACHES TO BREAK RESISTANCE

Resistance or cross‐resistance of neonicotinoids (IRAC MoA subgroup 4A) is more commonly due to CYP_450_ detoxification than to *n*AChR mutants or variants, as described for the Colorado potato beetle (*Leptinotarsa decemlineata*).^30^ For example, field‐collected strains of imidacloprid‐resistant brown planthoppers (BPH; *Nilaparvata lugens*) from China and India had enhanced levels of CYP_450_ activity.^31^ Likewise, enhanced monooxygenase activity was also associated with neonicotinoid resistance in strains of green peach aphids (*Myzus persicae*)^32^ and whiteflies (*Bemisia tabaci*).^33^

Recently, distinct high‐affinity binding sites for dinotefuran in the abdominal nerve cord of the American cockroach *Periplaneta americana* have been characterized.^34^

This difference in the imidacloprid and dinotefuran binding sites might be responsible, at least in part, for differences in the evolution of resistance to dinotefuran and other neonicotinoids in *P*. *americana*.

### 3.1 Nicotinic acetylcholine receptor competitive modulators

The global and enormous economic success of synthetic *n*AChR competitive modulators (IRAC MoA main group 4) as insecticides has been reviewed in various articles and book chapters over the past decade.^35^, ^36^

**Figure 7:**
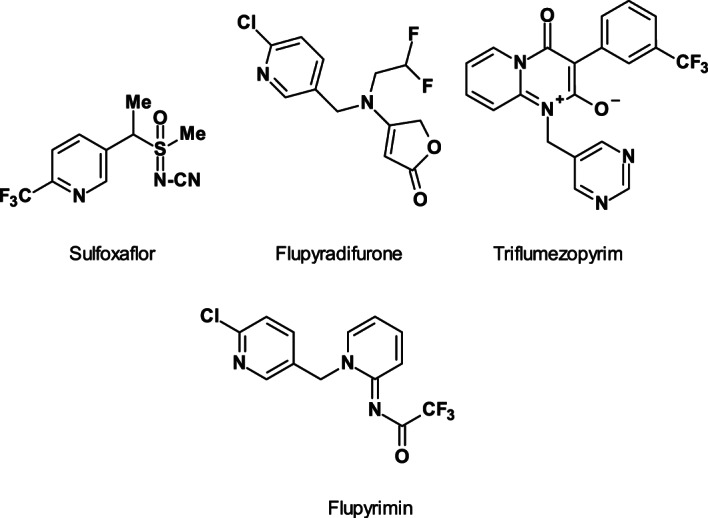
Structures of the fluorinated nAChR competitive modulators sulfoxaflor, flupyradifurone, triflumezopyrim and the developmental candidate flupyrimine.

Commercial use of fluorinated *n*AChR competitive modulators began in 2012 with the sulfoximines (sulfoxaflor, 2012; IRAC MoA sub‐group 4C) (Fig. 7) as the first subclass, followed by the butenolides (flupyradifurone, 2015; IRAC MoA sub‐group 4D) (Fig. 7) as second fluorine‐containing *n*AChR competitive modulators; the mesoionics (triflumezopyrim, 2018; IRAC MoA sub‐group 4E) (Fig. 7) complete the insecticides available as a third class.

Although these insecticides are believed to all have the same target site, current evidence indicates that the risk of metabolic cross‐resistance between sub‐groups 4A to 4E is low.^2^ In comparison with the neonicotinoids (IRAC MoA sub‐group 4A), commercialized fluorinated *n*AChR competitive modulators have lower or relatively low oral and contact toxicities to honey bees.

Flupyrimin (ISO provisionally approved, IRAC MoA not yet classified) (Fig. 7), a candidate of an older class, is under development, demonstrating potency to imidacloprid‐resistant rice pests together with superior safety toward pollinators.^37^

#### 3.1.1 *Sulfoximines*

The racemic sulfoxaflor (Fig. 7) is effective on a wide range of sap‐feeding insect strains that evolved metabolically based resistance. The lack of cross‐resistance to sulfoxaflor in these metabolically resistance strains seems to be due in large part to its chemical structure. Studies demonstrated that sulfoxaflor is not readily metabolized by the P_450_ monooxygenases such as CYP6G1 or CYP6CM1 often associated with resistance to neonicotinoids and insecticides in aphids, whiteflies, and other insects.^38^ The lack of an tetrahedral hybridized amine nitrogen in the sulfoxaflor molecule appears to limit susceptibility to metabolism by monooxygenases.^39^ Molecular modelling was used to investigate the interaction of neonicotinoids (e.g. imidacloprid) and sulfoxaflor in both wild‐type and target‐site resistant (R81T) *n*AChRs.^40^ Both imidacloprid and sulfoxaflor interact with arginine at position 81 of the green peach aphid (*M*. *persicae*) ß1 subunit of *n*AChR. But, the strength of this binding is far less for sulfoxaflor than for imidacloprid.

**Table 1:** Comparison of agonist activities of the metabolite *des‐*methyl‐broflanilide and fipronil according to membrane potential assay of wild‐type and mutant GABA_A_Rs and GlyRs

Receptor type	*Des*‐methyl‐broflanilide IC_50_ value (μm)	Fipronil IC_50_ value (μm)
Human (GABA_A_R) α1β2γ2	>3	0.29
Mammalian (GABA_A_R) α1β3γ2	>3	0.42
Human (GlyR) α1β	>3	0.815

Data partly taken from Nakao *et al*.^26^, based on data Zhao *et al*.^28^.

#### 3.1.2 *Butenolides*

Flupyradifurone (Fig. 7) controls neonicotinoid‐resistant whitefly (*Bemisia* sp.) populations.^41^ B‐ and Q‐type strains of cotton whitefly (*B*. *tabaci*) having > 1300‐ and 250‐fold resistance ratios against the neonicotinoid imidacloprid, exhibit threefold and sevenfold lower susceptibility to flupyradifurone, respectively when compared with a susceptible reference strain in leaf‐dip bioassays. It has been shown that flupyradifurone lacks cross‐resistance to neonicotinoids driven by monooxygenase enzymes such as CYP6CM1,^42^ a CYP_450_, highly overexpressed in cotton whitefly populations resistant to imidacloprid. CYP6CM1 overexpression is described as one of the major mechanisms of resistance to imidacloprid and pymetrozine (by hydroxylation), but not flupyradifurone.^43^

Based on recommended field rates, flupyradifurone results in good control of whitefly resistant to neonicotinoids (IRAC MoA subgroup 4A) worldwide (Table 2).

**Table 2:** Efficacy of flupyradifurone against neonicotinoid‐resistant whitefly species (*Bemisia* sp.) and neonicotinoid ‐tolerant greenhouse whitefly (*Trialeurodes vaporariorum*) in various countries

Species		Country	Efficacy^a^	Rate [g a.i./ha]
Neonicotinoid‐resistant	*Bemisia* sp.	Brazil	+++	90–125
	*B*. *argentifolii*	USA	+++	205
	*B*. *tabaci*	Spain	++++	112.5–150
	*B*. *tabaci*	Japan	+++	200–400
	*B*. *tabaci*	China	+++(+)	125–150
Neonicotinoid‐tolerant	*T*. *vaporariorum*	Italy	+++	112.5
	*T*. *vaporariorum*	Colombia	++(+)	270

^a^ ++++, excellent; +++, good; ++, satisfactory; +, marginal; −, insufficient; 0, no activity. Activities in between are indicated by parentheses. (Data taken from Jeschke *et al*.^41^).

An explanation could be that the butenolide pharmacophore moiety, in combination with the unique *N*‐2,2‐difluoroethyl residue in flupyradifurone increases its insecticidal activity and prevents oxidative metabolization by CYP6CM1. This exactly matches the experimental findings in bioassays and biochemical assays.

In addition, by creation of a homology model of sensitive aphid *n*AChR (*M*. *persicae*), it was found that flupyradifurone can interact with tyrosine (Tyr)‐residues from the α subunit in a ‘hydrogen‐bridge‐like fashion’ to the hydroxy function of these amino acids through its *N*‐2,2‐difluoroethyl unit.^44^ This hydrogen bridge‐like interaction between the fluorine atom in the fluorinated side chain of flupyradifurone as an acceptor and a hydroxy function of a tyrosine from the same loop in the α subunit as a donor is not possible for the other *n*AChR competitive modulators investigated.

#### 3.1.3 *Mesoionics*

The first member of the mesoionics group triflumezopyrim^45^ (Fig. 7), contains a 3‐(trifluoromethyl)‐phenyl moiety coupled with a mesoionic, dipolar core. Triflumezopyrim is active against susceptible and neonicotinoid‐resistant hopper species in Asia such as the rice BPH and whitebacked planthopper (*Sogatella furcifera*).

**Figure 8:**
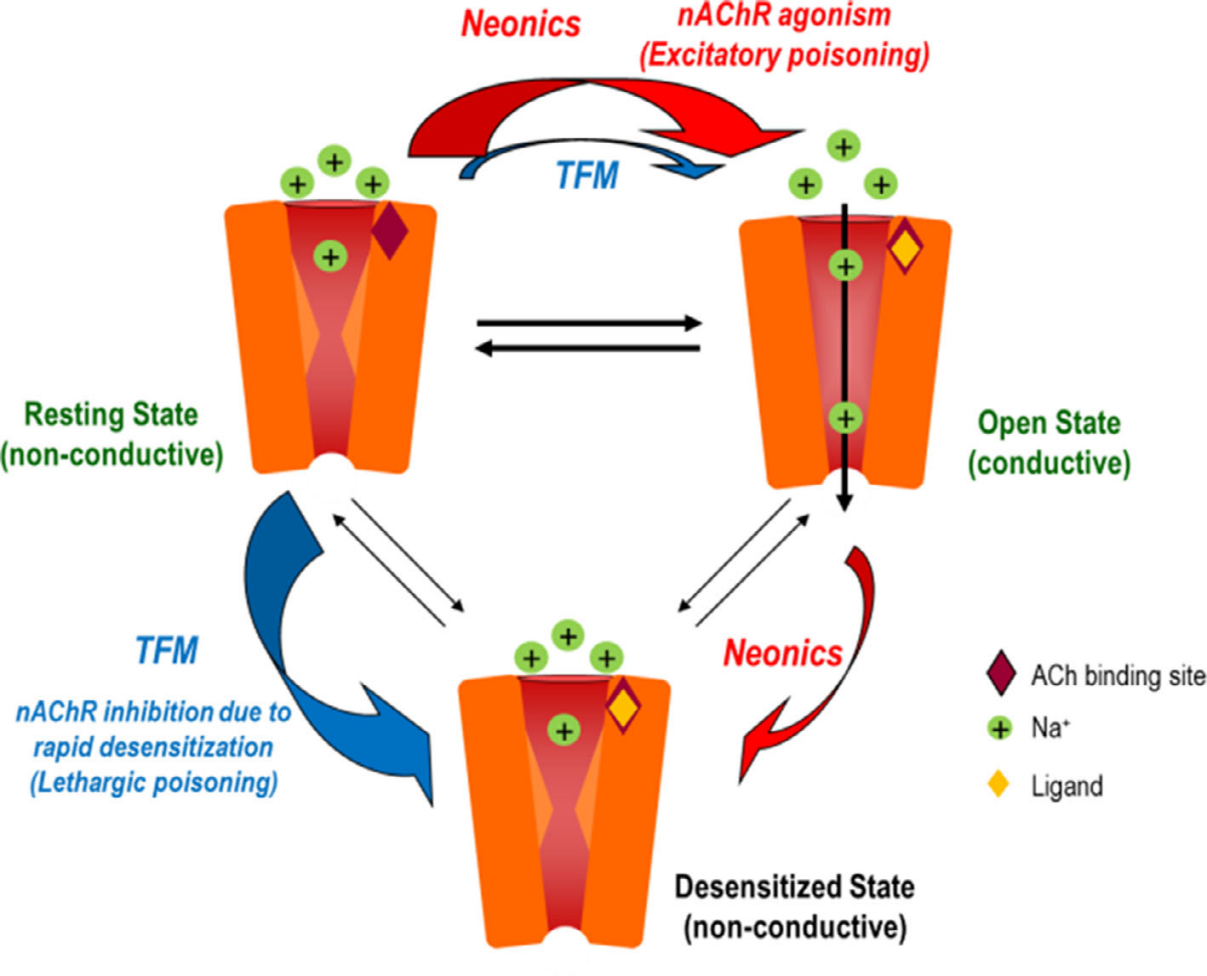
Mechanism of action for mesoionic insecticides. It is hypothesized that triflumezopyrim rapidly shifts the majority of *n*AChRs to a desensitized state (wide blue arrow) resulting in their inability to respond to activation by the endogenous neurotransmitter, acetylcholine. A small proportion of receptors which are non‐desensitizing (*n*AChN), remain available for activation (thin blue arrow) however this requires very high concentrations of triflumezopyrim. The net effect is reduced activity in the nervous system resulting in lethargic poisoning. In contrast, neonicotinoids activate both desensitizing (*n*AChD) and non‐desensitizing (*n*AChN) receptors. Although a number of receptors become desensitized (non‐conductive) a significant percentage become activated thereby producing excessive nerve activity and excitatory poisoning symptoms. (Figure was taken from D. Cordova *et al*.,^45^)

Given the rapid and prolonged *n*AChR inhibitory action, it was hypothesized that mesoionic insecticides such as triflumezopyrim induce a shift in the *n*AChR state from ‘closed/resting’ to ‘desensitized”, as shown in Fig. 8, resulting in a reduction of insect CNS activity, resulting in insect death.^46^

By contrast, submicromolar concentrations of neonicotinoids activate *n*AChRs. Although some receptors become desensitized (*n*AChD), a significant proportion shift to the open state, thereby causing nerve depolarization and excitatory poisoning.

Triflumezopyrim controls planthoppers through both ingestion and contact activity, and controls populations that have already demonstrated decreased sensitivity or even documented resistance to other important insecticidal classes, such as buprofezin (IRAC main group 16), fipronil (IRAC sub‐group 2B) and the neonicotinoids (IRAC sub‐group 4A) used for planthopper control.^47^

Triflumezopyrim is active against resistant populations of BPH, there is a lack of cross‐resistance between the imidacloprid (IRAC sub‐group 4A) and triflumezopyrim (IRAC sub‐group 4E). The comparative activity of triflumezopyrim and imidacloprid against field‐collected populations of imidacloprid‐resistant BPH from China is in the ratio 1:36.

Therefore, in future, the mesoionic insecticide will play a major role in IRM for planthopper control. In this context, recommendations^46^ have already been developed to delay resistance to triflumezopyrim, such as:

1. spraying this insecticide only once per season,
2. limiting its application to twice per year, and
3. mixing with an insecticide having a different MoA and following triflumezopyrim application ~ 3 weeks later.

### 3.2 Selected development candidates active nAChR competitive modulators

Flupyrimin (ISO provisionally approved, IRAC MoA not yet classified; Fig. 7) exhibits good potency against neonicotinoid‐insensitive rice insect pests and has superior safety toward pollinators.^37^

## 4 NEW PRODUCTS FOR SELECTED MODES OF ACTION

In addition to the currently most efficient MoAs affecting the insect CNS, insect muscle physiology is a common target for commercial products (Section 1, Fig.1). In this context, insect RyR is an insecticide target, which is essential for the muscle contraction and its importance is growing (Section 4.1).

Conversely, inhibition of lipid synthesis is currently the most important MoA for mite control.^48^ Currently, a small class of very potent ACCase inhibitors dominate the global acaricide market (Section 1) and further efforts are being made in the search for new a.i. (Section 4.2).

### 4.1 Ryanodin receptor modulators

Insects treated with highly specific RyR modulators (IRAC MoA main group 28) have similar symptoms to those treated with the toxic plant alkaloid ryanodine (extracted from *Ryania speciosa*). The insecticidal class of diamides (phthalic diamides and anthranilamides) act as such modulators.^7^, ^49^

**Figure 9:**
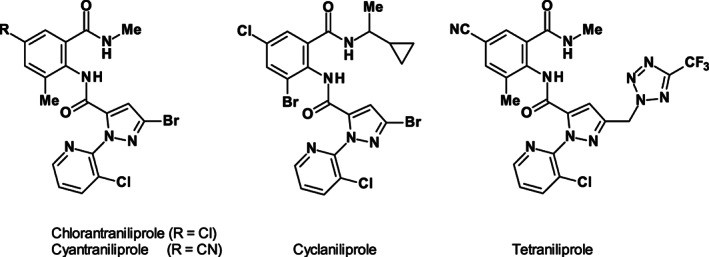
Structures of the RyR modulators chlorantraniliprole, cyantraniliprole, cyclaniliprole and tetraniliprole.

Inspired by the enormous success of the anthranilamides, chlorantraniliprole and cyantraniliprole as RyR modulators, the structural analogues cyclaniliprole and tetraniliprole were synthesized (Fig. 9).

The insecticide cyclaniliprole contains a 5‐bromo‐2‐(3‐chloro‐2‐pyridinyl)‐pyrazol‐3‐carboxyl moiety, but differs from chlorantraniliprole (R = Cl) in both the phenyl substitution (Br instead of methyl) and the amide residue (*rac*‐CO‐NH‐CHMe‐cyclopropyl instead of CO‐NH‐methyl). Cyantraniliprole^50^ is active against lepidoptera, leafminers, whiteflies and others, and seems to be active against resistant strains RyR(G4946E) of diamondback moth (*P*. *xylostella*). It can be used as an insecticide on apples, pears, peaches, apricots, nectarines, plums, grapes (table and wine) and potatoes, and on glasshouse tomatoes, peppers and aubergines.^51^

Tetraniliprole contains a heterocycle 5‐trifluoromethyl‐2*H*‐tetrazol‐2‐yl‐methyl linked by a methylene unit as replacement of bromine.

Tetraniliprole has good activity against a broad spectrum of insect pests including lepidoptera, coleoptera, leafminer, and selected other Diptera and aphids. It can be applied as foliar and soil treatment from early to late season. In comparison with the known RyR modulators chlorantraniliprole and cyantraniliprole, the novel tetraniliplole has a good profile against insect pests such as apple codling moth (*Cydia pomonella*), peach fruit moth (*Carposina sasakii*), citrus leafminer (*Phyllocnistis citrella*) and the European grapevine moth (*Lobesia botrana*) (Table S2).

Current progress in elucidating the structure of mammalian RyRs, together with molecular characterization of diamide‐intensive mutations of insect RyRs has resulted in a better understanding of their molecular MoA. Based on their broad‐spectrum activity against insect pests, target specificity, and low mammalian toxicity, diamides have been well suited to both IRM programs and IPM strategies.

**Figure 10:**
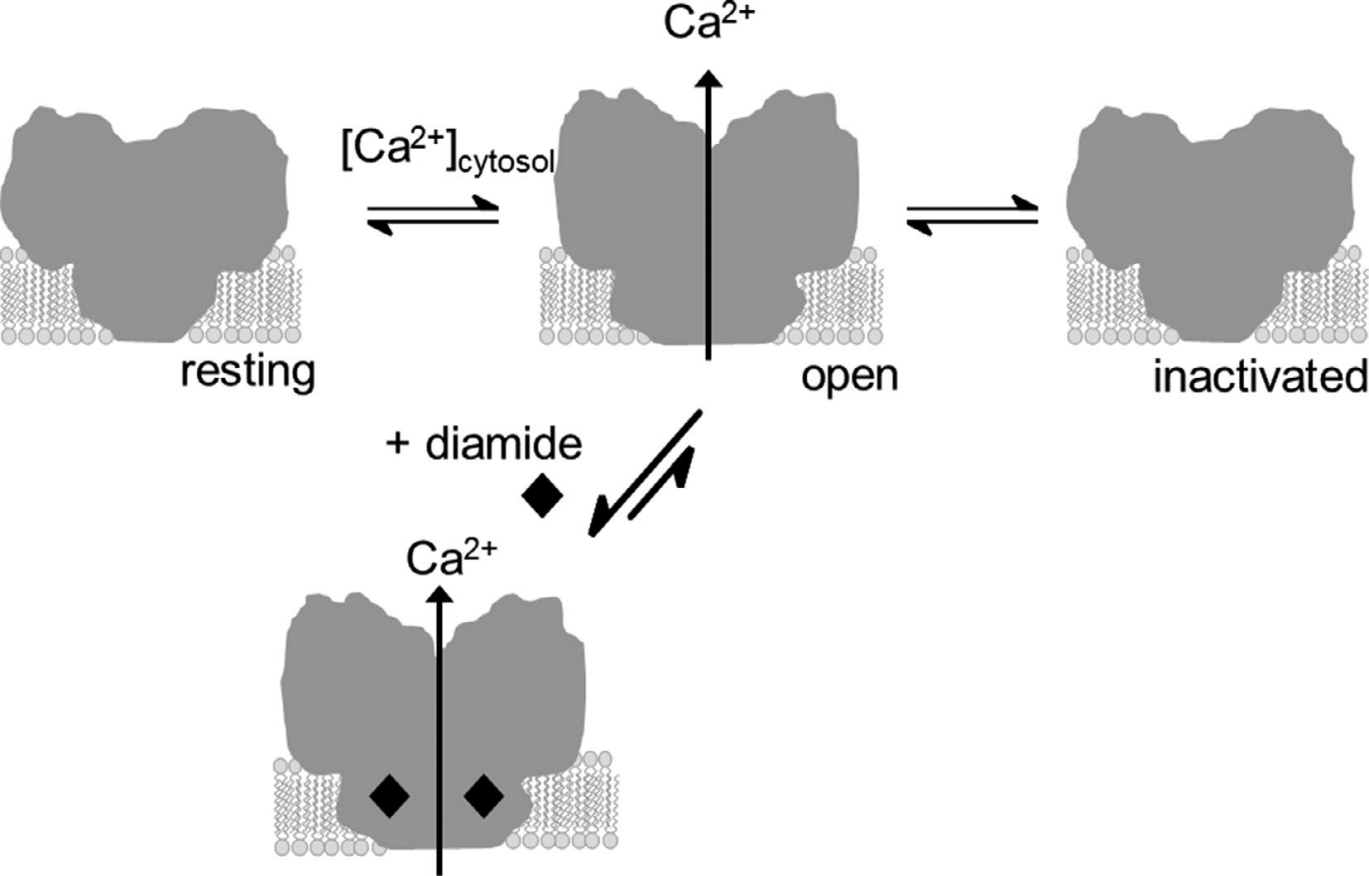
Hypothesis of the diamide mechanism of action. The equilibrium between resting state and open states is regulated by the intracellular calcium concentration. Diamides bind reversibly and selectively to the calcium‐conducting state thereby shifting the equilibrium towards open channel conformation. The inactivated conformation has been implied by structural studies of the rabbit RyR1. Its physiological significance is not clear. Adapted fromU. Ebbinghaus‐Kintscher *et al*.*^9^*.

In the view of the diamide MoA, it is relevant that the conformational states (two state model: open *versus* closed) seem to be associated with specific ligand‐binding properties.^52^ In terms of the conformational selection model,^53^ it is conceivable that diamide binding may shift the conformational equilibrium of insect RyRs toward the open state, thereby increasing the fraction of calcium‐conducting channels in the endoplasmic reticulum/sarcoplasmic reticulum (ER/SR) membrane arrays of muscle cells.^54^ (Fig. 10).

In recent years, target‐site mutations conferring resistance to both phthalic diamides (flubendiamide) and anthranilamides (chlorantraniliprole, cyantraniliprole) have evolved in diamondback moth (*P*. *xylostella*) populations from China and Southeast Asia.^55^

### 4.2 Inhibitors of lipid synthesis: acetyl‐CoA carboxylase inhibitors

ACCase (ACC, EC 6.4.1.2) is a biotinylated enzyme that plays a fundamental role in the metabolism of fatty acids by catalyzing carboxylation of acetyl‐CoA to malonyl‐CoA using ATP as a source of energy.^56^

Three so‐called pro‐insecticidal ‘cyclic ketoenoles’ (KTEs) have been commercialized, the two tetronic acid derivatives spirodiclofen and spiromesifen and the tetramic acid derivative spirotetramat (IRAC MoA main group 28).

**Figure 11:**
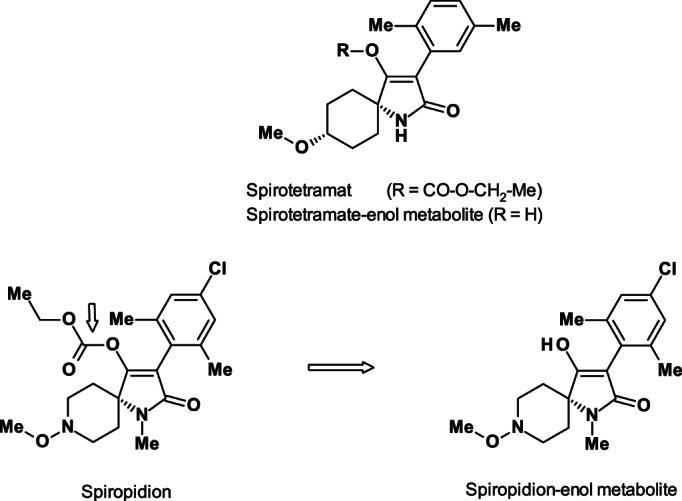
Structures of the pro‐insecticides spirotetramat and spiropidion and their insecticidally active metabolites spirotetramat‐enol and spiropidion‐enol.

Spiropidion (ISO provisionally approved) is in development as a fourth KTE.^57^ Compared with spirotetramat (R = CO‐O‐CH_2_‐Me; Fig. 11), the new *N*‐methyl tetramic acid derivative spiropidion contains a spiro *N*‐methoxy piperidine moiety instead of the spiro methoxy cyclohexane moiety in spirotetramat (replacement of CH by N in the spirocyclic 6‐ring). The resulting pro‐insecticide has a broad range of activity against sucking pests because of the ambimobile behavior (translaminar distribution and two‐way xylem/phloem mobility) of the spiropidion‐enol in the same way as spirotetramat‐enol (R = H; Fig. 11).

**Figure 12:**
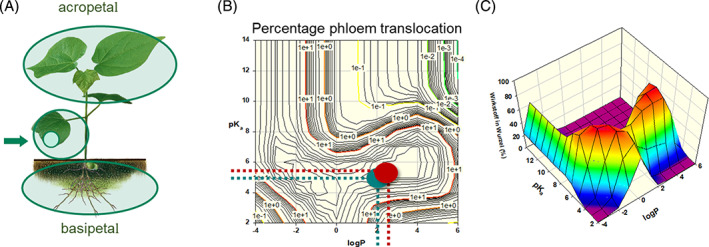
Distribution of the pro‐insecticides spirotetramat and spiropidion in the whole plant after foliar application (A: green arrow = position of application). The simulation model (B, C) reflexes the current knowledge on phloem translocation and can predict the mobility of most compounds. Both insecticidally active metabolites spirotetramat‐enol (B: interrupted line and point in green) and spiropidion‐enole (B: interrupted line and point in brown) demonstrate their ambimobile behavior (translaminar distribution and two‐way xylem/phloem mobility, good retention in phloem) the vascular system of the plant. The treatment of mature leaves gives full protection of new growth. *Source:* Data of Walter Schmitt (personal communication) adapted from the mathematical model on D. A. Kleier, 1988; C = 3D view of the diagram).

Spiropidion is also quickly transformed in leaves after foliar application *in planta* hydrolysis to the insecticidally active spiropidion‐enol, which enters the vascular system of the plant and is acropetal symplastly and basipetal translocated, protecting the whole plant (Fig. 12a). A result is the protection of young, untreated leaves and roots in whole cabbage plants.^57^ Similar to spirotetramat‐enol (p*K*_a_ = 5.2), spiropidion‐enol has suitable physico‐chemical properties such as being a weak acid with a p*K*_a_ value of 5.53, which results in phloem mobility (Fig. 12b,c). The weak acidic and lipophilic properties of the enol metabolites define their retention in phloem and long distance movement.

The simulation model based on Kleier,^58^ predicts comparable good phloem mobility which is confirmed by biological activity. Both spirotetramat‐enol and spiropidion‐enol arrive in the xylem and phloem, and are retained there.

Spiropidion exhibits low contact activity but good systemic movement in the plant. It can be used via flexible foliar and soil applications in field crops, specialist crops and vegetables, controlling a broad range of sucking pests such as aphids, whiteflies, scales and mites, and an IPM compatibility may be expected.

## 5 NEW INSIGHTS INTO KNOWN MECHANISMS OF ACTION

In the past 5 years, new insights have been gained into known compounds classified by IRAC into former main group 9 (Section 5.1). This is applicable for the reclassified bifenazate (Section 5.2) and for the molecular target site of thiazolidinone‐type acaricides that also affects the target site of benzoylphenylureas (BPUs) exemplified by flufenoxuron (Section 5.3).

### 5.1 Changes in the former IRAC main group 9

Investigation into the MoAs of ‘selective homopteran feeding blockers’ (IRAC main group 9) such as pymetrozine and flonicamid, led to the finding that both interact specifically with chordotonal organs at an unidentified target site.^59^ Pymetrozine and pyrifluquinazone (Fig. S1) perturb chordotonal organ function by specifically modulating the Nan‐Iav TRPV channels.^60^ In addition, flonicamid (Fig. S1) also modulates chorodontonal organs but appears to act distinctly.

Based on these findings, the IRAC has made the changes to the MoA classification scheme:^61^

1. renaming IRAC main group 9 as ‘Chordotonal organ TRPV channel modulators’, which includes pymetrozine and pyrifluquinazone in sub‐group 9B;
2. assigning flonicamid (formerly IRAC sub‐group 9C) to IRAC main group 29 ‘Chordotonal organ modulators – undefined target site’, which do not bind to the Nan‐Iav TRPV channel complex (Section 2.2).

### 5.2 Reclassification of bifenazate

In 2016, the IRAC reclassified bifenazate (Fig. S2), one of the acaricides most frequently used to control spider mites, from category unknown (UN) to chemical class category 20D, defined as MET III inhibitors.^62^, ^63^

Despite first assumptions that bifenazate is neurotoxic at GABA receptors, genetic evidence has pointed towards a mitochondrial target site, in particular the Q_o_ site of mitochondrially encoded cytochrome *b*.^64^ Furthermore, no mutations linked with resistance in GABA receptors were found, and their expression level was not changed overall between strains.

### 5.3 Molecular target site of thiazolidinone‐type acaricides

Recent findings using high‐resolution genetic mapping have shown that resistance to the thiazolidinone hexythiazox (Fig. S3) is monogenetic, recessive. Resistance in two‐spotted spider mites (*T*. *urticae*) strain (HexR) is due to a target‐site mutation of CHS1 conferring cross‐resistance to clofentezine and etoxazole (Fig. S3).^65^ This suggests CHS1 as the molecular target site of thiazolidinone‐type acaricides.^66^

The findings demonstrate a shared molecular MoA for the structurally diverse mite growth inhibitors hexythiazox, clofentezine and etoxazole as inhibitors of an essential, non‐catalytic activity of CHS1.^67^

Given previously documented cross‐resistance between hexythiazox, clofentezine, and the benzoyl phenyl urea flufenoxuron in the field for the red spider mite (*Panonychus ulmi*),^68^ CHS1 should be also considered a potential target site of insecticidal benzoyl phenyl ureas.^69^

In this context, and to minimize the likelihood of further evolution of resistance, sequential use of hexythiazox, clofentezine, and etoxazole to control mite populations should be avoided when implementing IRM strategies.

## 6 PROMISING NEW CHEMISTRY

**Figure 13:**
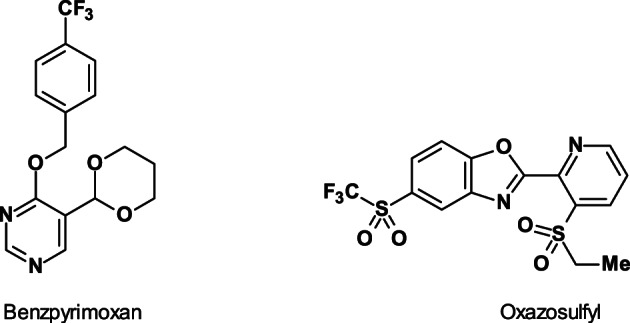
Insecticide development candidates benzpyrimoxan and oxazosulfyl.

Benzpyrimoxan (ISO provisionally approved, IRAC MoA not yet classified; Fig. 13)^70^ is an extremely promising hopper insecticide with high nymphicidal activity against rice plant hoppers such as BPH (*N*. *lugens*) as well as whitebacked planthopper (*S*. *furcifera*), and a low impact on non‐target organisms including pollinators and beneficial arthropods.

Benzpyrimoxan is highly active, specifically against the nymphal stages, and demonstrates molting inhibition different from that of other existing insect growth regulators. Its insecticidal activity against nymphs is very superior to that against other stages and the field biological performance at 50–75 g a.i. ha^−1^ revealed a favorable environmental profile without any resurgence and with high activity even against BPH (*N. lugens*) that had evolved resistance to major chemical classes of insecticide.^70^

Oxazosulfyl (ISO provisionally approved, IRAC MoA not yet classified; Fig. 13) is a novel sulfyl insecticide (F_3_C‐SO_2_‐group) possessing potent and broad‐spectrum insecticidal activity, including against Hemiptera, Coleoptera, and Lepidoptera, for insect control in rice fields.^71^

In the 1990s, Zeneca described special substituted chiral azabicyclo[3.3.1]nonanes that have insecticidal activity against peach aphid (*M*. *persicae*).^72^

**Figure 14:**
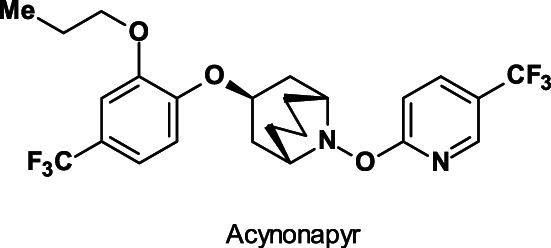
Acaricide development candidate acynonapyr.

Acynonapyr (ISO provisionally approved, IRAC MoA not yet classified; Fig. 14), an acaricide with a new substituted azabicyclo[3.3.1]nonane core, is useful for control of spider mites in vegetables, tea, and citrus fruits.^72^

## 7 CONCLUDING REMARKS AND PROSPECTS

Numerous older agrochemicals have been removed from the marketplace because their profiles no longer meet current standards. The diminished number of available acaricides and insecticides results in fewer options for resistant management. The innovative acaricides and insecticides launched between 2008 and 2020 support the search for agrochemicals, as reflected by the novel MET II inhibitor pyflubumide, the Nan‐Iav TRPV channel inhibitor afidopyropen and the GABA‐gated chloride channel allosteric modulators broflanilide and fluxametamide. Interestingly, the viable pro‐pesticide concept was successfully applied for both the acaricide pyflubumide and the insecticide broflanilide, which readily undergo *in vivo* transformation in mites or insects to give biologically active metabolites.

Continuing research of highly specific RyR modulators resulted in the discovery of cyantraniliprole and tetraniliprole with partially improved insecticidal activity.

In a similar way, the lipid synthesis and growth regulation of insects has been further studied resulting in the ACCase inhibitor spiropidion (ISO provisionally approved), which will soon be on the market as a fourth cyclic KTE. Transformation of the pro‐insecticide spiropidion by *in planta* hydrolysis, results in formation of the spiropidion‐enol metabolite, demonstrating the ambimobile behavior of this ‘weak acid’ in the plant.

The latest generation of modern agrochemicals, including those in the development pipeline, again illustrate that the impact of halogens remains significant (e.g. *meta*‐diamides). In this context, the influence of fluorine‐containing substituents such as trifluoromethyl or 2,2‐difluoroetyl on the resistance‐breaking properties of insecticides can be exemplified by the new generation of fluorinated *n*AChR competitive modulators.

Finally, the number of agrochemicals containing asymmetric centers, and selected examples of chiral agrochemicals in development have shown that chirality in modern crop protection products is increasing and gaining in importance (e.g. afidopyropen, arylisoxazolines).

In future, modern agricultural chemistry will continue to build innovative solutions against all globally important arthropod pests (mites and insects) in crops for customers that address pest control today and tomorrow—resulting in low impact on beneficial organisms and ecosystems, higher crop yields, and better food quality.

## Supporting information


**Figure S1.** Structures of pymetrozine, pyrifluquinazone and flonicamid.
**Figure S2.** Structures of MET III inhibitor bifenazate.
**Figure S3.** Structures of hexythiazox, clofentezine and ethoxazole.

**Table S1.** Classification of mode of action for current and potential new commercial acaricides and insecticides according to IRAC discussed in this review.

**Table S2.** Field performance of the tetraniliprole compared with the antranilamides chlorantraniliprole and cyantraniliprole used as market standards.
