# Supplementary material for: Status and outlook for acaricide and insecticide discovery
Source: Pest Manag Sci. 2020 Sep 28;77(1):64–76. doi: 10.1002/ps.6084 (PMC7756306; doi:10.1002/ps.6084)
Supplement: Supplementary file 1 — Figure S1. Structures of pymetrozine, pyrifluquinazone and flonicamid. Figure S2. Structures of MET III inhibitor bifenazate. Figure S3. Structures of hexythiazox, clofentezine and ethoxazole. [file PS-77-64-s001.doc]

**SUPPORTING INFORMATION**

**Figure S1.** Structures of pymetrozine, pyrifluquinazone and flonicamid.

**Figure S2.** Structures of MET III inhibitor bifenazate.

**Figure S3.** Structures of hexythiazox, clofentezine and ethoxazole.
