# Supplementary material for: Status and outlook for acaricide and insecticide discovery
Source: Pest Manag Sci. 2020 Sep 28;77(1):64–76. doi: 10.1002/ps.6084 (PMC7756306; doi:10.1002/ps.6084)
Supplement: Supplementary file 2 — Table S1. Classification of mode of action for current and potential new commercial acaricides and insecticides according to IRAC discussed in this review. [file PS-77-64-s002.docx]

**SUPPORTING INFORMATION**

**Table S-1.** Classification of MoAs for current and potential new commercial acaricides and insecticides according to IRAC discussed in this review.

| **IRAC Group** | **IRAC Main-group** | **Primary site of action** | **IRAC Sup-group^a^** | **Class** | **Active ingredient or new-term product** | **Target** | **Launch Year** | **Section** | **Comment** |
| --- | --- | --- | --- | --- | --- | --- | --- | --- | --- |
| 4 | Nicotinic acetylcholine receptor (*n*AChR) competitive modulators | Nerve action | 4C  4D  4E | Sulfoximines  Butenolides  Mesoionics | Sulfoxaflor  Flupyradifurone  Triflumezopyrim | *n*AChR  *n*AChR  *n*AChR | 2012  2015  2018 | 3.1.1  3.1.2  3.1.3 |  |
| 9 | Chordotonal organ TRPV channel modulators | Nerve action | 9B | Pyridine azomethine derivatives | Pymetrozine  Pyrifluquinazon | Chordotonal organ TRPV channel | 1993  2010 | 5.1  5.1 | Target defined |
|  |  |  | 9D | Pyropenes | Afidopyropen | Chordotonal organ TRPV channel | 2018 | 2.2 |  |
| 10 | Mite growth inhibitors affecting CHS1 | Growth regulatiuon | 10A | Clofentezine  Hexythiazox | Clofentezine  Hexythiazox | CHS1  CHS1 | 1983  1985 | 5.3  5.3 | Target defined |
|  |  |  | 10B | Etoxazole | Etoxazole | CHS1 | 1998 | 5.3 |  |
| 15 | Inhibitors of chitin biosynthesis affecting CHS1 | Growth regulation | 15 | Benzoylureas | *e.g.* Flufenoxuron | CHS1 | 1989 | 5.3 | Target defined |
| 20 | Mitochondrial complex III electron transport inhibitors | Enery metabolism | 20D | Bifenazate | Bifenazate | Complex III | 1999 | 5.2 | Reclassified in 2016 |
| 23 | Inhibitors of acetyl CoA carboxylase | Lipid synthesis, growth regulation | 23 | Tetramic acid derivatives | Spiropidion | ACCase | Not yet | 4.2 |  |
| 25 | Mitochondrial complex II electron transport inhibitors | Energy metabolism | 25B | Carboxanilides | Pyflubumide | Complex II | 2014 | 2.1 |  |
| 28 | Ryanodine receptor (RyR) modulators | Nerve and muscle action | 28 | Diamides | Cyclaniliprole  Tetraniliprole | RyR | 2017  2019 | 4.1  4.1 |  |
| 29 | Chordotonal organ Modulators – undefined target site | Nerve action | 29 | Flonicamid | Flonicamid | Chordotonal organ | 2004 | 5.1 | New class defined |
| 30 | GABA-gated chloride channel allosteric modulators | Nerve and muscle action | 28 | Isoxazolines  *Meta*-diamides | Fluxametamide  Broflanilide | GABA | 2018  2019 | 2.3.1  2.3.3 |  |

**Table S-1.** (continuation).

| **IRAC Group** | **IRAC Main-group** | **Primary site of action** | **IRAC Sup-group^a^** | **Class** | **Active ingredient or new-term product** | **Target** | **Launch Year** | **Section** | **Comment** |
| --- | --- | --- | --- | --- | --- | --- | --- | --- | --- |
| 32 | Nicotinic Acetylcholine Receptor (nAChR) Allosteric Modulators – Site II | Nerve action | 32 | GS-omega/ kappa HXTXHv1a peptide | GS-omega/kappa HXTX-Hv1a | *n*AChR | 2018 | 2.4 | New Target defined |

^a^ Cited from Nauen et al.^25^ and IRAC (<http://www.irac-online.org>) MoA Classification Version 9.4, March 2020. TRPV = transient receptor potential cation channels ("V" for vanilloid), CHS1 = chitin synthase 1, GABA = gamma-aminobutyric acid.
