# Supplementary material for: Status and outlook for acaricide and insecticide discovery
Source: Pest Manag Sci. 2020 Sep 28;77(1):64–76. doi: 10.1002/ps.6084 (PMC7756306; doi:10.1002/ps.6084)
Supplement: Supplementary file 3 — Table S2. Field performance of the tetraniliprole compared with the antranilamides chlorantraniliprole and cyantraniliprole used as market standards. [file PS-77-64-s003.doc]

**SUPPORTING INFORMATION**

**Table S-2.** Field performance of tetraniliprole compared to the antranilamides chlorantraniliprole and cyantraniliprole used as market standards.

| **Crop** | **Pest Species** | **Tetraniliprole** | **Efficacy^a^**  **Chlorantraniliprole** | **Cyantraniliprole** |
| --- | --- | --- | --- | --- |
| Apple | *Cydia pomonella* | ++++ | +++ | ++++ |
| Peach | *Carposina sasakii* | ++++ | +++ | ++ |
| Citrus | *Phyllocnistis citrella* | ++++ | +++ | +++ |
| Citrus/StF | *Ceratitis capitata* | +++ | n.d. | +++ |
| Grape | *Lobesia botrana* | ++++ | ++ | n.d. |
| Stone/SoF | *Drosophila suzukii* | +++ | + | +++ |
| Coffee | *Leucoptera coffeelia* | ++++ | ++++ | ++++ |

^a^ ++++ excellent, +++ good, ++ satisfactory, + marginal, - insufficient, 0 no activity. StF = Stone Fruit, SoF = Soft Fruit. (Source: Bayer AG)
